# Antiretroviral Therapy and Viral Suppression Among Foreign-Born HIV-Infected Persons Receiving Medical Care in the United States: A Complex Sample, Cross-Sectional Survey

**DOI:** 10.1097/MD.0000000000003051

**Published:** 2016-03-18

**Authors:** Tanya R. Myers, Xia Lin, Jacek Skarbinski

**Affiliations:** From the Division of Healthcare Quality and Promotion (TRM); and Division of HIV/AIDS Prevention (XL, JS); and Epidemic Intelligence Service (XL), Centers for Disease Control and Prevention, Atlanta, GA.

## Abstract

Immigrants to the United States are more likely to be diagnosed with human immunodeficiency virus (HIV) infection compared with native-born persons. Navigating access to healthcare in the United States can be challenging for foreign-born persons, and HIV treatment outcomes may be suboptimal for these persons. We compared characteristics of and assessed disparities in clinical outcomes of foreign-born persons in care for HIV in the United States.

The Medical Monitoring Project is a complex sample, cross-sectional survey designed to be nationally representative of HIV-infected adults receiving medical care in the United States.

Using data from 2009, 2010, and 2011, we conducted descriptive analyses and multivariable logistic regression to assess associations between foreign-born status and antiretroviral therapy (ART) prescription, and between foreign-born status and viral suppression.

In all, 13.4% of HIV-infected persons were self-identified as foreign-born; the most common regions of birth were Central America and Mexico (45.4%) and the Caribbean (16.0%). Nearly 90% of foreign-born persons were diagnosed with HIV after entry into the United States. Compared with US-born persons, foreign-born persons were more likely to be younger, Hispanic, less educated, and uninsured. The prevalence of ART prescription (prevalence ratio 1.00; 95% confidence interval 0.98–1.02) was not significantly different between foreign-born and US-born persons. A higher percentage of foreign-born persons achieved viral suppression compared with US-born persons (prevalence ratio 1.05; 95% confidence interval 1.00–1.09).

No major disparities in ART prescription and viral suppression were found between foreign-born and US-born HIV-infected persons receiving medical care, despite higher percentages being uninsured.

## INTRODUCTION

In the United States, immigrants are more likely to be diagnosed with human immunodeficiency virus (HIV) infection than native (US-born) persons,^1^ but very little is known about the quality of medical care and clinical outcomes among foreign-born HIV-infected persons living in the United States. In general, foreign-born persons in the United States are less likely to access routine healthcare, less likely to have health insurance, and report lower levels of satisfaction with the healthcare received.^2^ Retention of HIV-infected immigrants in care may be especially challenging for those living in marginalized communities, those emigrating from countries where HIV denialism is prevalent, and those whose social or religious communities view HIV infection as a moral fault. United States immigration policies classified HIV-positive status as an inadmissible condition for entry of immigrants and refugees from 1991 to 2009.^3^ Although a diagnosis of HIV infection was eliminated as an exclusionary condition in 2010, fear of negative repercussions from diagnosis or disclosure of HIV status within foreign-born populations may remain. Lowered access to, utilization of, and satisfaction with healthcare provision can lead to suboptimal clinical outcomes for any HIV-infected person; these factors may be especially impactful on clinical outcomes for new immigrants.

The few comparisons of clinical outcomes between foreign-born and US-born have been limited to specific geographic regions or ethnic/racial groups. These studies have found similar proportions of foreign-born persons receiving antiretroviral therapy (ART) as US-born.^4–6^ Most have not observed statistically significant differences in measures of disease progression or viral suppression.^4,5,7^ Another study classifying subjects by documented citizenship or alien status found no differences in viral suppression between undocumented Hispanics receiving care as compared to documented Hispanics and whites.^8^ While local studies are important for assessing regional health care provision and needs, these analyses have limited sample sizes for foreign-born and may have been insufficiently powered to detect differences between populations. Additionally, 2 studies that have explored time of diagnosis (before or after migration) were limited to specific regions.^5,9^

We used data from a survey specifically designed to be nationally representative of HIV-infected persons receiving medical care to describe characteristics of foreign-born HIV-infected persons receiving medical care and to explore clinical outcomes of ART receipt and viral suppression achieved by foreign-born HIV-infected persons as compared with their US-born counterparts. This study provides the first national-level description of foreign-born HIV-infected persons in care in the United States and an evaluation of clinical outcomes of foreign-born persons as compared with US-born persons on a national level.

## METHODS

The Medical Monitoring Project (MMP) is a surveillance system designed to produce nationally representative, cross-sectional estimates of behavioral and clinical characteristics of HIV-infected adults receiving medical care in the United States.^10^ MMP utilizes a 3-stage, complex sampling design in which US states and territories are sampled, followed by facilities providing outpatient HIV medical care in those jurisdictions, then HIV-infected adults (aged 18 years and older) receiving care in those facilities. We used data from 2009, 2010, and 2011 MMP data collection cycles, including information on adults with at least 1 HIV medical care visit to participating facilities during January to April 2009 (2009 cycle), January to April 2010 (2010 cycle), and January to April 2011 (2011 cycle). Data were collected from June 2009 through May 2012 using face-to-face or telephone interviews and medical record abstractions. MMP protocols are publicly available, and further details regarding sampling locations and methodology are available in yearly surveillance summaries.^10–12^ The MMP data sets are not publicly available.

The Centers for Disease Control and Prevention's National Center for HIV/AIDS, Viral Hepatitis, STD, and TB Prevention has determined the MMP to be a nonresearch public health surveillance activity used to guide disease control programs and policy; therefore, it was not reviewed by a federal institutional review board. Participating facilities obtained local institutional review board approvals as required, and informed consent was obtained from all interviewed participants.

All sampled states and territories participated in the MMP. Facility response rates were 76% (461/603) in 2009, 81% (474/582) in 2010, and 83% (473/570) in 2011. Approximately 50% of persons sampled from these facilities completed an interview and had their medical records abstracted (4217/9038 in 2009, 4474/9300 in 2010, and 4503/9023 in 2011), with 13194 patients sampled over 3 cycles. Patients were not excluded from sampling if they had participated in previous data-collection cycles (3.5% in 2010 participated in 2009, 1.6% in 2011 participated in 2010, and 3.0% in 2011 participated in 2009 or 2010). Foreign-born status was missing for 4 patients; these patients were excluded from the analysis, leaving a total of 13190 patients in our dataset. A specific country of origin was missing for an additional 2 patients who were self-identified as foreign-born; these patients were included in analyses except when region of origin was a variable.

### Measures

Two outcome variables were explored independently: prescription of ART and achieving viral suppression. Prescription of ART was defined as any record of ART prescription in the 12 months before interview. Viral suppression was defined as undetectable or <200 copies/mL for the most recent measure. The main exposure variable was foreign-born status. Participants were considered foreign-born if they listed a country or territory of birth other than the United States or Puerto Rico. Geographic regions were defined as per recommendations of the United Nations Statistics Division, with Central America inclusive of Mexico.^13^ No imputation was made for refusals to provide information on country of birth. Covariates included sociodemographic characteristics and self-reported variables of time since HIV diagnosis, healthcare coverage, age at migration (if foreign-born), time in the United States since migration, sexual risk behavior, drug and alcohol use, homelessness and incarceration, and racial/ethnic category.

Time of diagnosis (before or after migration) was calculated using self-reported time since diagnosis and time in the United States. Service needs were defined as unmet if at least 1 ancillary service need was unmet in the past year. Consistent care was defined as receipt of 2 or more CD4+ T-lymphocyte cell (CD4) count measures within the past year; this measure was chosen to align with the standard of care over the time period of data collection in this study. Case management service need was defined as unmet if service was needed but not received during the past year. Poverty level was defined using standard federal thresholds based on yearly income and number of household dependents.^14^

### Statistical Analysis

The MMP collected information on all sampled patients and facilities, including sex, age, race, length of time since diagnosis, and facility's HIV patient load, and was able to compare respondents and nonrespondents. Data were then weighted according to these analyses to minimize nonresponse bias.^15^ All analyses accounted for complex sample design and unequal selection probabilities by using survey procedures in SAS 9.3 (SAS Institute, Inc., Cary, NC) and SUDAAN 10.0.1 (RTI, Research Triangle Park, NC).

Absolute numbers and weighted frequencies were calculated for demographic, clinical, and behavioral characteristics. Associations between characteristics and foreign-born status were evaluated using a Rao-Scott chi-square test. Logistic regression analyses were conducted independently for both outcomes of interest (ART prescription and viral suppression). Because certain characteristics were only applicable to foreign-born persons (region of birth, age at migration, diagnosis before or after migration, and time in the United States) and these characteristics might moderate the effect of being foreign-born, separate logistic regression analyses were conducted for the association between each outcome variable and foreign-born status, categorized by these characteristics.

Final multivariable models were developed separately for each binary outcome, ART prescription, and viral suppression. Current age, sex, and race/ethnicity were chosen as a priori potential confounders because we expected these might be related to both foreign-born status and outcomes. Race/ethnicity was not included as a potential confounder in analyses in which foreign-born were categorized by region, because region of origin will, in some cases, define race/ethnicity (eg, people migrating from Mexico would likely describe themselves as Hispanic/Latino). Additional variables, including education level, poverty level, healthcare coverage, time since diagnosis, receipt of consistent care, categorization of disease severity, identified sexual risk factors, unmet service needs, and unmet case management needs were selected as potential confounders if associated with both the outcome for that model and the exposure for that model (based on Rao–Scott chi-square test, *P* ≤ 0.05). Our criterion for a confounder to be included in the final multivariable model was any potential confounder that caused 10% or more change on the association (measured by prevalence ratio). A priori variables were not forced into models if they did not meet this criterion. Univariate logistic regression models using the binary foreign born variable as the exposure of interest and either ART or viral suppression as the outcome proceeded to multivariable logistic regression models whether crude associations were significant (*P* < 0.10) or not. Independent models were also constructed for foreign-born as a nonbinary variable, with separate models to consider foreign-born by region of birth, age at migration, diagnosis before or after migration, and time in the United States; for these models, multivariable logistic regression models were only constructed if crude associations were significant (*P* < 0.10).

## RESULTS

Of all HIV-infected adults receiving medical care in the United States, 13.4% (95% confidence interval [CI] 11.7–15.0) were foreign-born. A total of 126 unique countries or territories were identified. Mexico was most frequently listed, followed by Cuba and Haiti (Table 1). An estimated 29% of foreign-born HIV-infected persons originated from Mexico, with far fewer originating in Cuba and Haiti (5% from each). Countries of birth were also grouped by region and are displayed in Table 2 along with other characteristics specific to foreign-born persons. Together, the Central American (including Mexico) and Caribbean regions accounted for 61% of foreign-born HIV-infected persons receiving medical care in the United States. The majority (74%) of foreign-born persons were aged 18 or older at migration. Most foreign-born persons had resided in the United States for at least 10 years (83%), and nearly 90% were diagnosed with HIV after entering the United States.

**TABLE 1:**
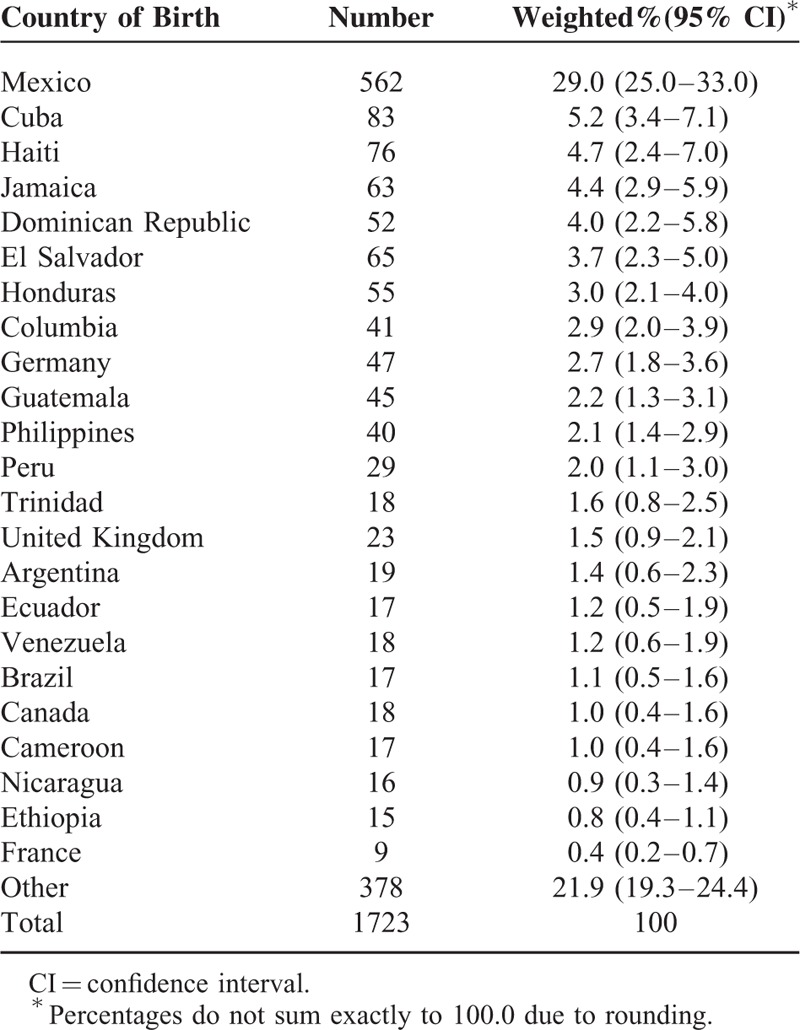
Countries of Birth Reported by Foreign-born HIV-infected Adults Receiving Medical Care in the United States (Medical Monitoring Project, 2009–2011)

**TABLE 2:**
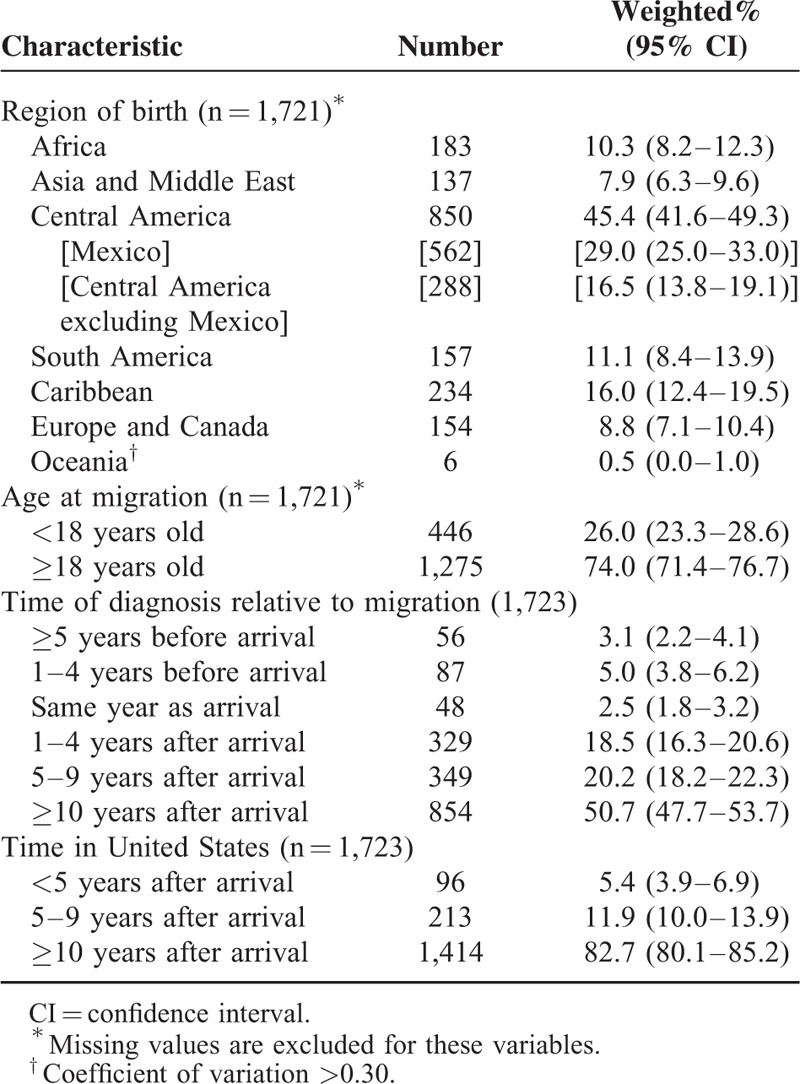
Characteristics of Foreign-born HIV-infected Adults Receiving Medical Care in the United States (Medical Monitoring Project, 2009–2011)

Demographic, clinical, and behavioral characteristics of foreign-born and US-born persons are listed in Table 3 . Foreign-born persons were younger, more often Hispanic, and less well-educated than US-born persons. Foreign-born persons were more likely to be uninsured, more recently diagnosed with HIV, more likely to have evidence of consistent care and be adherent to ART, and more likely to have achieved viral suppression than US-born persons. Lower proportions of foreign-born persons reported having been homeless or incarcerated in the past 12 months. Foreign-born persons were significantly less likely to have had sex without a condom or to have used drugs (injection or noninjection) in the past year, and were less likely to have used alcohol excessively in the past 30 days. Foreign-born persons were also significantly less likely to have been diagnosed with major depression. There were no significant differences between foreign-born and US-born persons in receipt of ART prescription, stage of disease, or geometric mean CD4 count category in the past 12 months.

**TABLE 3:**
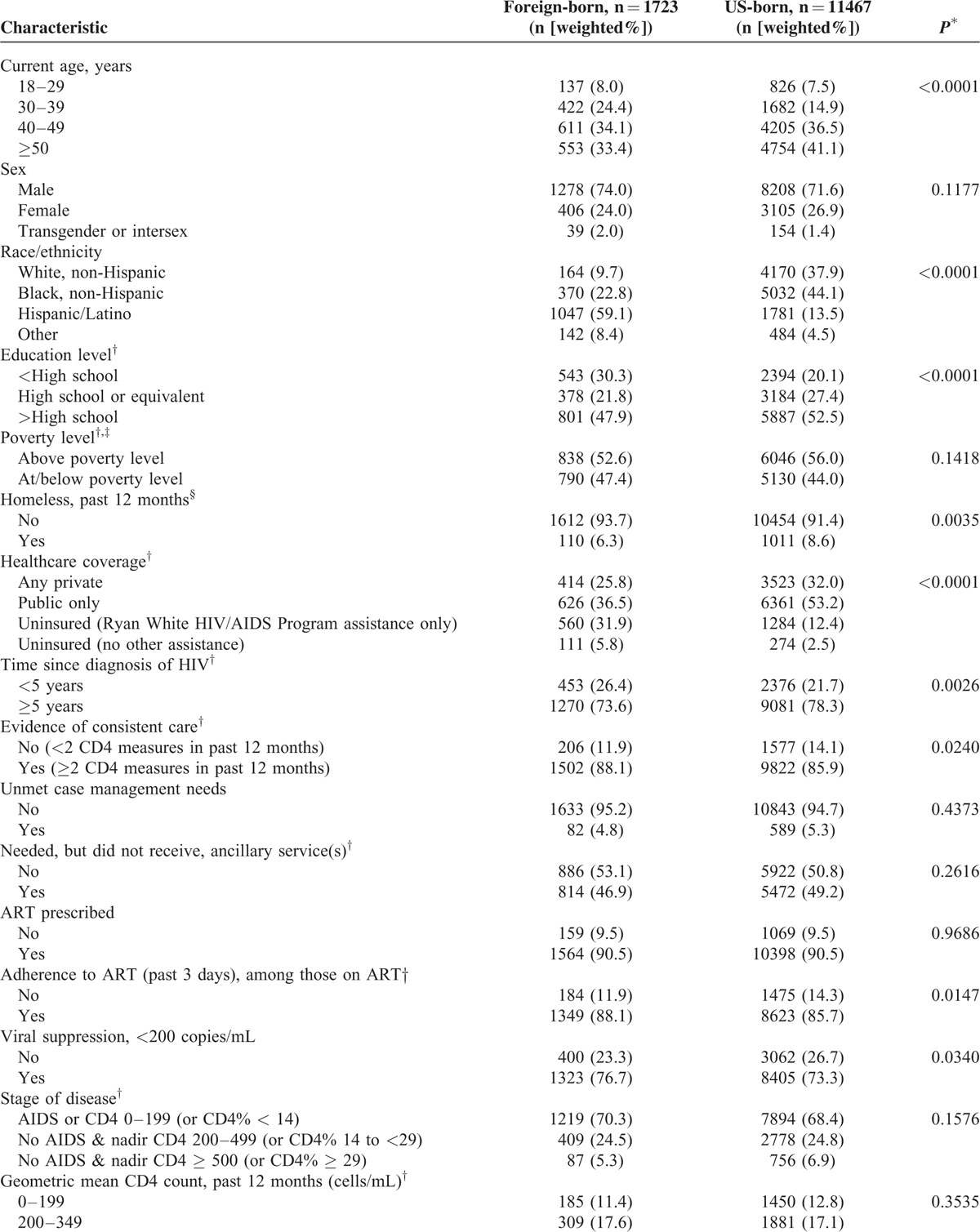
Comparison of Foreign-born Versus US-born HIV-infected Adults Receiving Medical Care in the United States (Medical Monitoring Project, 2009–2011)

**TABLE 3 (Continued):**
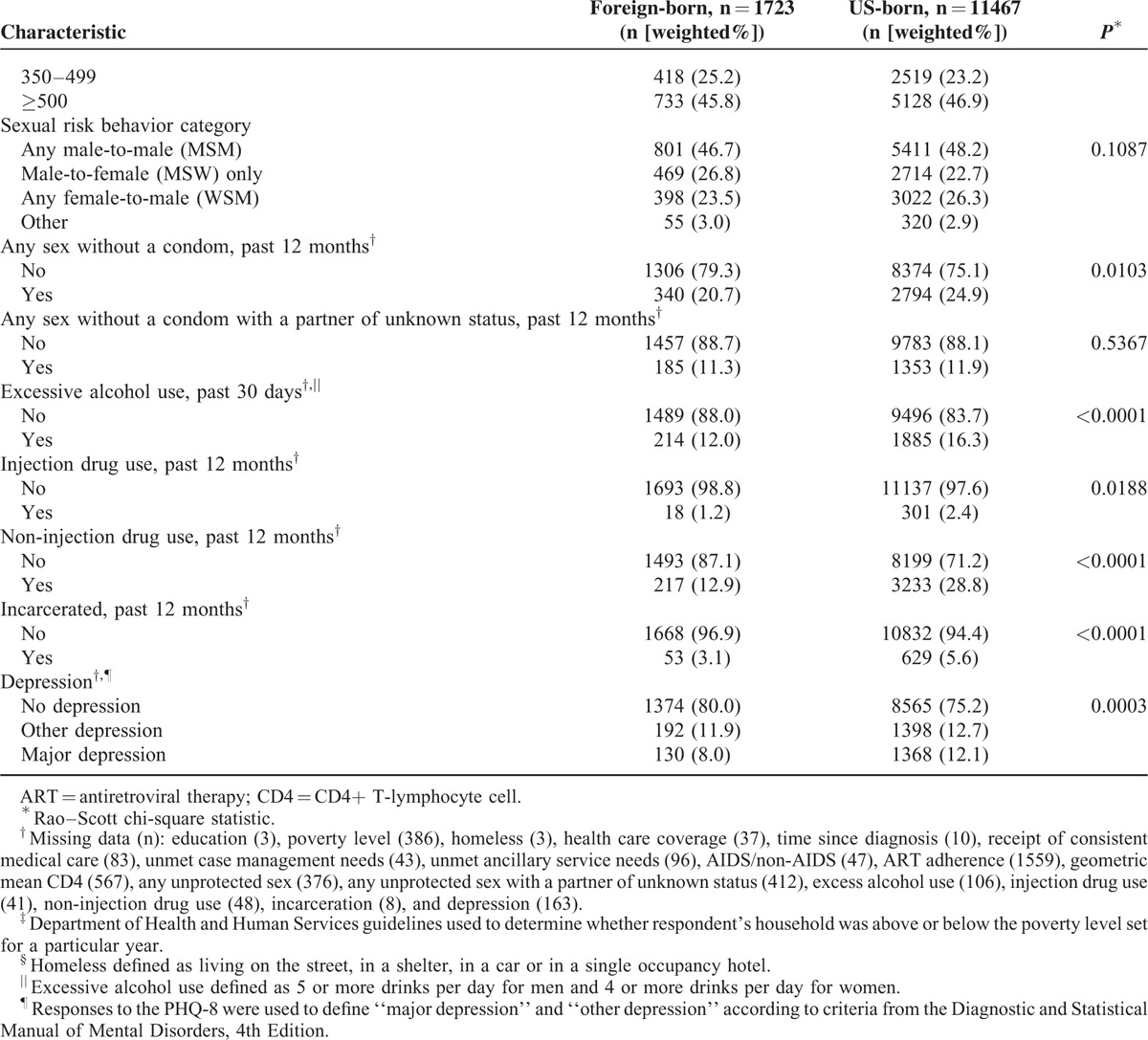
Comparison of Foreign-born Versus US-born HIV-infected Adults Receiving Medical Care in the United States (Medical Monitoring Project, 2009–2011)

### Receipt of ART Prescription

Prevalence ratios (PRs) assessing the association of foreign-born status and receipt of ART prescription are shown in Table 4. In the simplest model with foreign-born included as a binary exposure variable, there was no evidence that receipt of ART prescription differed between foreign-born and US-born patients. Evaluation of confounders for inclusion in a multivariable model failed to find any variable meeting the prespecified criteria. Four additional separate models characterized foreign-born persons by characteristics thought to moderate the effect of being foreign-born: region of birth, age at migration, location of diagnosis (before or after migration), and time in the United States. None of these models achieved the significance level required to pursue multivariable modeling. Similar to the simplest model, there was no evidence in these additional models that receipt of ART prescription differed between foreign-born and US-born patients.

**TABLE 4:**
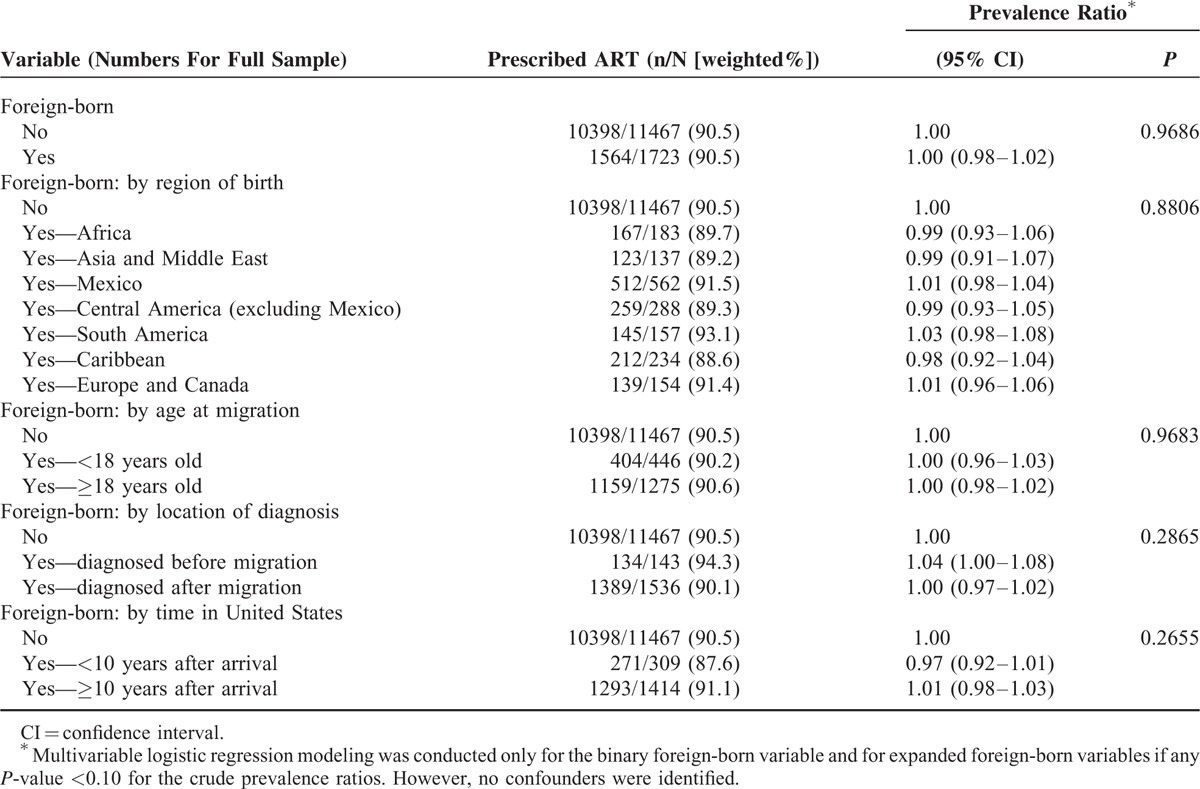
Associations Between Receipt of Antiretroviral Therapy (ART) Prescription and Foreign-born Status Among HIV-infected Adults Receiving Medical Care in the United States (Medical Monitoring Project, 2009–2011)

### Viral Suppression

Prevalence ratios for assessing the association of foreign-born status and viral suppression are shown in Table 5. When confounders were evaluated for each model, none met prespecified criteria for inclusion, and therefore, all PRs presented in Table 5 are unadjusted. In the simplest model comparing foreign-born (as a binary variable) to US-born persons, slightly higher proportions of foreign-born persons achieved viral suppression as compared with US-born persons. Significant differences were also found in models where foreign-born persons were categorized by region of birth, age at migration, and time in the United States. In the model categorized by region of birth, a significantly higher proportion of foreign-born persons from Mexico achieved viral suppression (adjusted PR = 1.12; 95% CI 1.06–1.19; *P* = 0.02) as compared with US-born persons or those migrating from other regions. When foreign-born persons were categorized by age at migration, those who migrated as adults (≥18 years of age) achieved viral suppression in significantly higher proportions than US-born or those who migrated at younger ages (adjusted PR = 1.06; 95% CI 1.01–1.10; *P* = 0.05). Similarly, foreign-born persons living in the United States for 10 or more years achieved viral suppression in higher proportions than their US-born counterparts or those more newly arrived in the United States (adjusted PR = 1.06; 95% CI 1.01–1.10; *P* = 0.04).

**TABLE 5:**
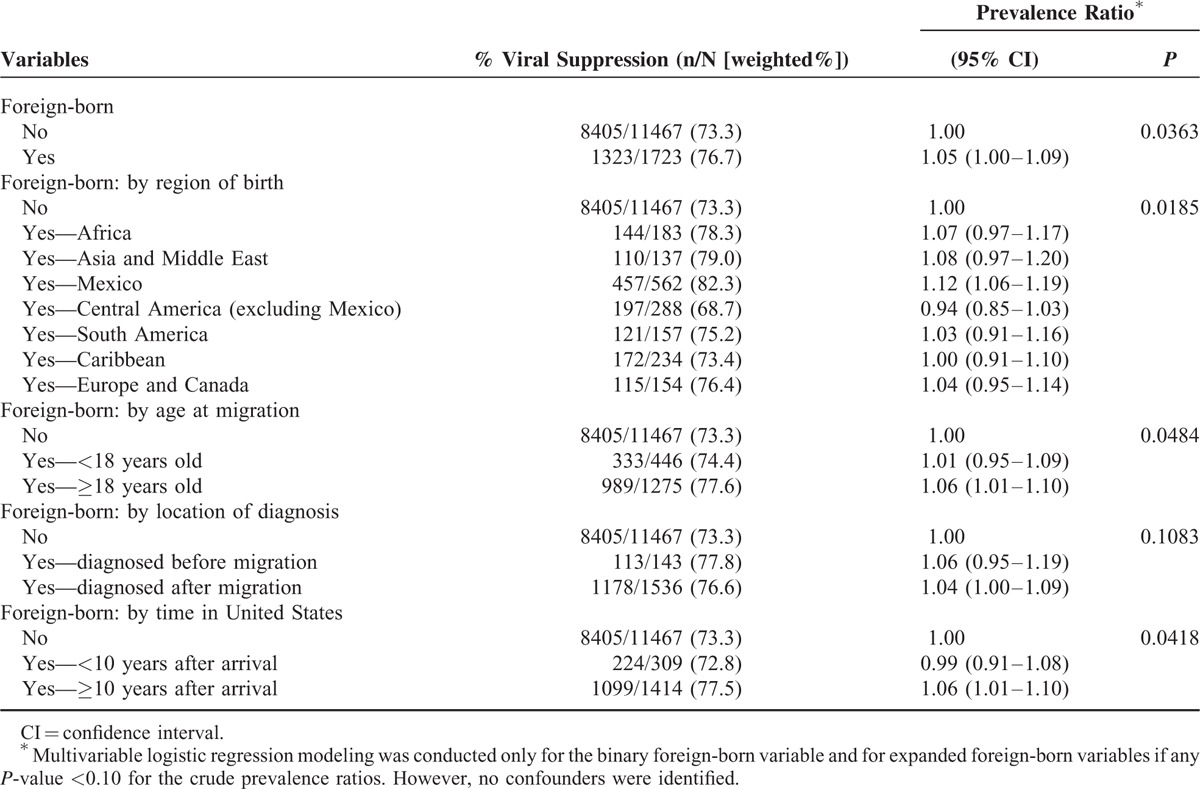
Associations Between Foreign-born Status and Viral Suppression Among HIV-infected Adults Receiving Medical Care in the United States (Medical Monitoring Project, 2009–2011)

## DISCUSSION

This analysis presents the first nationally representative characterization of HIV-infected foreign-born adults receiving medical care in the United States and the first to directly characterize time of diagnosis as before or after migration. There was no evidence that on a national level, foreign-born persons are prescribed ART either more or less than US-born persons; this result was consistent in all models. A slightly higher proportion of foreign-born persons achieved viral suppression as compared with US-born persons. Additionally, several subgroups achieved viral suppression in slightly higher proportions, including foreign-born persons who migrated as adults, foreign-born persons who had lived in the United States for 10 or more years, and foreign-born persons from Mexico.

The most recent American Community Survey (ACS) estimated that 12.9% of the total population of the United States are foreign-born persons, with the majority (65.3%) having lived in the United States for less than 10 years,^16^ as compared with the 13.4% foreign-born in the HIV-infected patient population in this study, with a minority (17.3%) having lived in the United States for less than 10 years. Our foreign-born participants reflect the multiplicity of countries from which immigrants and migrants arrive in the United States, although foreign-born for some regions were over or under-represented as compared with national estimates for foreign-born persons residing in the United States. Among the MMP participants, 45.4% were born in Central America (including 29.0% from Mexico), 16.0% in the Caribbean, 10.3% in Africa, 7.9% in Asia, and 11.1% in South America. Foreign-born HIV-infected persons from Mexico represented a proportion of HIV-infected individuals in MMP, which was similar to the ACS estimate that 29.3% of all foreign-born living in the United States are from Mexico; higher percentages of foreign-born persons from the Caribbean and Africa and lower percentages of foreign-born persons from Asia and the Middle East were observed in the MMP as compared with the ACS.^16^

General demographic features of foreign-born HIV-infected adults receiving medical care in our study are consistent with previous reports from specific regions of the United States and from origin-specific groups of foreign-born HIV-infected persons. Similar to previous reports, we found that foreign-born persons were younger than their US-born counterparts,^4,6,7^ less well-educated,^5^ less likely to report injection drug use,^7^ and less likely to have health insurance.^5,6^ We did not see differences in the proportions of male and female foreign-born HIV-infected patients compared with those who were US-born. Findings in previous studies seem to be influenced by region of birth of the study population. For example, in populations with mixed origins or in which foreign-born originated exclusively from Africa, foreign-born persons infected with HIV were less likely to be male than US-born persons infected with HIV.^4,7,17,18^ However, in Hispanic/Latino populations, foreign-born persons infected with HIV were more frequently male.^5,8^ Additionally, we did not find any differences in the proportions of foreign-born and US-born persons who reported male-to-male sexual contact as a mode of exposure to HIV; this finding is in contrast to numerous studies reporting that foreign-born persons infected with HIV are less likely to report male-to-male sexual contact as a mode of exposure.^4–7,17–19^ We believe that our analysis, based on a nationally representative sample of HIV-infected adults, addresses some of the limitations inherent to generalizing results from studies conducted in geographically limited populations in the United States, such as in single cities or states.

We found some key differences in our study population as compared with the demographic characteristics of the general population of foreign-born persons based on data from the ACS.^16^ Higher proportions of foreign-born HIV-infected persons were male (74.0% vs 49.1%), living at or below poverty level (47.4% vs 18.8%), and uninsured (37.7% vs 34.3%) as compared with foreign-born persons in the general US population. These differences might reflect factors that increase a person's risk of HIV infection (eg, males are more likely to acquire HIV than females in the United States) or might be associated with living with HIV infection (eg, loss of income and employer-based insurance due to disability related to HIV infection). Educational attainment levels were similar between foreign-born HIV-infected adults and foreign-born adults in the general US population.

Although viral suppression is achieved at higher proportions in older age groups within the United States,^20^ we did not find that age was a confounder in our models of viral suppression. Yet, foreign-born persons were younger than native-born persons in our study and achieved viral suppression in slightly higher proportions than native-born persons. This suggests that other unmeasured characteristics not related to age may lead to these slightly better clinical outcomes in foreign-born persons. Those who self-select for migration may have (or acquire during migration) particular skills that are related to the skills required to successfully navigate health systems, adhere to therapy, and maintain health-seeking behaviors after diagnosis with HIV.

The act of migration may place some immigrant populations at higher risk of HIV infection than they would have faced in their home countries.^21^ However, few studies have been able to definitively assess the proportion of HIV-infected persons who are diagnosed pre versus postmigration. Two studies of Hispanic/Latino immigrants have estimated, based on time reported in the United States and average time from infection to diagnosis, that HIV infection in this group of foreign-born persons is mainly due to HIV exposure after migration.^22,23^ Two studies have made estimates from individual-level data. Dennis et al^5^ found that 6.9% of southeastern-based Hispanic and Latino immigrants in care for HIV in their study were diagnosed before arrival in the United States, a figure similar to our estimate of 8.1%. For foreign-born persons residing in New York City and diagnosed with HIV during 2006 to 2012, Wiewel et al^9^ determined that 23% had probably acquired HIV outside the United States. Although our study evaluated location of diagnosis rather than location of acquisition, our results are consistent with that of Wiewel et al, considering lag time between infection and diagnosis. We estimated that 29.1% of foreign-born persons were diagnosed either before arrival or within the first 4 years in the United States.

We are encouraged by the findings that no significant differences were found in proportions of foreign-born persons who are in medical care and receiving ART as compared with US-born persons (either in aggregate or in the analyses by subgroups), and that foreign-born persons in medical care are achieving viral suppression in proportions similar to or better than US-born persons. These results suggest that after foreign-born persons navigate channels required to gain access to health care, they do not face significant risk of having worse outcomes than their US-born counterparts.

Our study is unique in that the overall sample was larger than most studies of HIV-infected foreign-born persons and had minimal risk of bias due to incomplete or inaccurate reporting (4 participants lacked data for foreign-born status, 2 did not identify country of birth). Participants were sampled through healthcare providers and were not excluded from participating in multiple cycles; however, given the low percentages of repeat sampling in the 2010 and 2011 cycle years, we expect minimal bias from inclusion of repeat participants. Results are representative only of foreign-born persons receiving care for HIV. The study was not designed to explore how all foreign-born persons infected with HIV compare with all native-born persons infected with HIV.

Weighting was used to adjust for nonresponse, but bias may still have occurred if foreign-born and US-born persons who refused to participate differed on characteristics related to outcomes and covariates included in the analysis. Unique reasons that foreign-born persons might refuse to participate could include language barriers, cultural concerns, or fears of threat to immigration status. MMP avoids asking for information that might reveal undocumented status in an effort to prevent the latter bias. Second, the structure of sampling may have led to decreased probability of selection for patients who are intermittent consumers of healthcare. Some groups likely to be more transitory in care are newly arrived foreign-born and undocumented foreign-born persons. The effect of these potential biases would have been to bias results toward the null hypothesis of no difference between foreign-born and US-born persons, as the foreign-born persons enrolled in the MMP would have been more acculturated and thus similar to US-born persons.

In a nationally representative sample of HIV-infected adults receiving medical care in the United States, we found no disparities in ART prescription between foreign-born HIV-infected persons compared with their US-born counterparts, despite the finding that foreign-born persons were more likely to be living at or below the poverty level and uninsured. Additionally, we found that foreign-born persons achieved viral suppression at similar or higher proportions than US-born persons. These findings provide reassurance that current programs (such as the Ryan White HIV/AIDS program) aimed at improving the HIV continuum of care through provision of services to people without adequate healthcare coverage or financial resources have been effective at addressing these needs for foreign-born persons. Finally, 9 in 10 HIV-infected foreign-born persons among this sample of persons receiving HIV medical care were diagnosed with HIV after arrival in the United States, indicating that the risk for HIV acquisition among foreign-born persons immigrating to the United States mainly occurs postmigration. HIV prevention programs should continue to provide services to foreign-born persons to reduce their risk of HIV infection.
